# Direct Reuse of Spent Nd–Fe–B Permanent Magnets

**DOI:** 10.3390/ma18132946

**Published:** 2025-06-21

**Authors:** Zara Cherkezova-Zheleva, Daniela Paneva, Sabina Andreea Fironda, Iskra Piroeva, Marian Burada, Maria Sabeva, Anna Vasileva, Kaloyan Ivanov, Bogdan Ranguelov, Radu Robert Piticescu

**Affiliations:** 1 Institute of Catalysis, Bulgarian Academy of Sciences, Acad. G. Bonchev St., Bldg. 11, 1113 Sofia, Bulgaria; daniela@ic.bas.bg (D.P.); mariasabeva.r@gmail.com (M.S.); anna@ic.bas.bg (A.V.); kaloyan@ic.bas.bg (K.I.); 2 National Research and Development Institute for Nonferrous and Rare Metals—IMNR, 178-184 Biruintei Blvd., Ilfov County, 077145 Pantelimon, Romania; sabina.munteanu@imnr.ro (S.A.F.); mburada@imnr.ro (M.B.); rpiticescu@imnr.ro (R.R.P.); 3 Institute of Physical Chemistry, Bulgarian Academy of Sciences, Acad. G. Bonchev St., Bldg. 11, 1113 Sofia, Bulgaria; ipiroeva@ipc.bas.bg (I.P.); rangelov@ipc.bas.bg (B.R.)

**Keywords:** critical and strategic resource materials, rare-earth elements, EoL Nd–Fe–B permanent magnets, mechanochemistry, waste processing, anisotropic Nd_2_Fe_14_B material preparation

## Abstract

Nd–Fe–B permanent magnets are vital for numerous key technologies in strategic sectors such as renewable energy production, e-mobility, defense, and aerospace. Accordingly, the demand for rare earth elements (REEs) enormously increases in parallel to a significant uncertainty in their supply. Thus, research and innovative studies are focus on the investigation of sustainable solutions to the problem and a closed-loop value chain. The present study is based on two benign-by-design approaches aimed at decreasing the recycling loop span by preparing standardized batches of EoL Nd–Fe–B materials to be treated separately depending on their properties, as well as using mechanochemical method for waste processing. The previously reported benefits of both direct recycling and mechanochemistry include significant improvements in processing metrics, such as energy use, ecological impact, technology simplification, and cost reduction. Waste-sintered Nd–Fe–B magnets from motorbikes were collected, precisely sorted, selected, and pre-treated. The study presents a protocol of resource-efficient recycling through mechanochemical processing of non-oxidized sintered EoL magnets, involving the extraction of Nd_2_Fe_14_B magnetic grains and refining the material’s microstructure and particle size after 120 min of high-energy ball milling in a zirconia reactor. The recycled material preserves the main Nd_2_Fe_14_B magnetic phase, while an anisotropic particle shape and formation of a thin Nd/REE-rich layer on the grain surface were achieved.

## 1. Introduction

### 1.1. Rare Earth Elements Rank Among the Most Critical and Strategic Resource Materials

**Figure 1:**
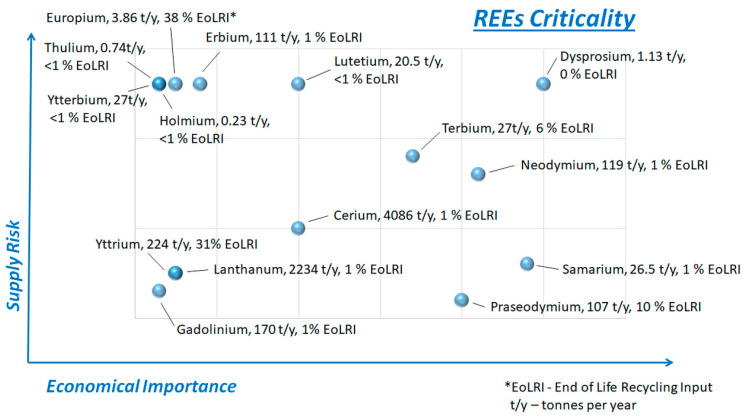
Criticality of REEs according to the current CRMs list [4], consumption and recycling rate for each rare earth element in 2023 according to the published statistics [6].

Critical and strategic resource materials (CRMs&SRMs) are vital to various industries due to their unique properties that are challenging to replicate using up-to-date technology and conventional methods [1,2,3]. CRMs&SRMs play a crucial role in modern technology, quality of life, and clean technology initiatives [3,4]. Rare earth elements (REEs) are some of the most critical materials on both the CRMs list [4] and the SRMs list [5]. Figure 1 illustrates their criticality according to the current CRMs list [4], together with the REE consumption of each element (measured in tons per year, t/y) and its recycling rate (end-of-life recycling input, EoLRI) in 2023 according to the published statistic for Europe according to EUROSTAT [6]. The respective values of the reported data for the United States are very close, according to the National Minerals Information Center of the US government [7]. REEs are irreplaceable in applications such as solar panels, wind turbines, electric batteries, electric vehicles, automotive catalysts, energy-efficient lighting, mobile phone and computer displays, glass additives, metallurgy, and more. However, their supply can face commercial scarcity and challenges due to delivery restrictions for economic or geopolitical reasons, increased regional demand, or concerns related to corporate social responsibility. Issues such as conflicts and human rights violations, forced community displacement, unsafe labor conditions, environmental impacts, and waste management contribute to these challenges for multinational companies [1,2]. Due to the small number of countries that dominate the REE supply chain, numerous concerns have been raised about stability, geopolitical risks, and price fluctuations [6,7]. This instability creates challenges for manufacturers in terms of cost forecasting. Now, Europe is intensifying its investigations into increasing the possibilities of producing rare earth elements, both from primary and secondary sources across the continent [8,9]. Recent statistical data extracted from [6] and presented in Figure 1 show that the reported enormously high REE consumption can be sustainable only in case of increased resource efficiency, i.e., significantly raising the current very low recycling rate of end-of-life REE-containing materials.

### 1.2. Nd–Fe–B Magnet’s Role in Green Energy and Carbon Reduction Strategy

**Figure 2:**
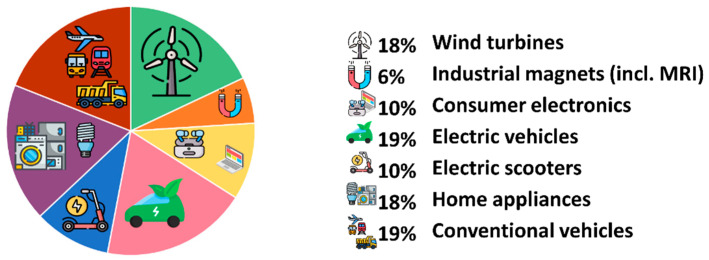
Nd–Fe–B demand shares in various industrial sectors in the EU. The electric scooter sector has one of the largest market shares. The data are based on estimation by Rizos et al. [15].

The neodymium–iron–boron magnet, recently called “the magnet that made the modern world” [10], has become the most widely used type of permanent magnet in many high-performance applications since its independent invention by Masato Sagawa and John Croat in the early 1980s. Today, Nd–Fe–B magnets are at the heart of green energy and carbon reduction strategies, accounting for nearly 97% of the market [4,5,6,7]. These magnets are smaller and lighter than other types of magnets, and this property makes them extremely appropriate in applications where space and weight are important, e.g., electric motors and electrical equipment [8,9,10,11]. The diverse applications of REEs in driving industrial advancements, together with the respective consumption shares of REEs, are illustrated in Figure 2. One of the major drivers for increased Nd–Fe–B magnet consumption is the transition towards electrification in the automotive industry, predominantly the manufacture of electric vehicles (EVs) and hybrid vehicles (HVs) [7,8,10,11]. Nd–Fe–B magnets are employed in propulsion motors, which contribute to better efficiency and performance of electric automobiles [11,12,13]. In addition, due to their high magnetic power and small size, Nd–Fe–B magnets are extensively used in a variety of consumer electronics, including mobile phones, laptops, and audio devices, and are useful for applications such as speakers, motors, and sensors [6,13,14,15]. Thus, the electric scooter segment holds one of the largest market shares, and it is going to continue its dominance [14,15].

### 1.3. Recycling of Nd–Fe–B Magnets

**Figure 3:**
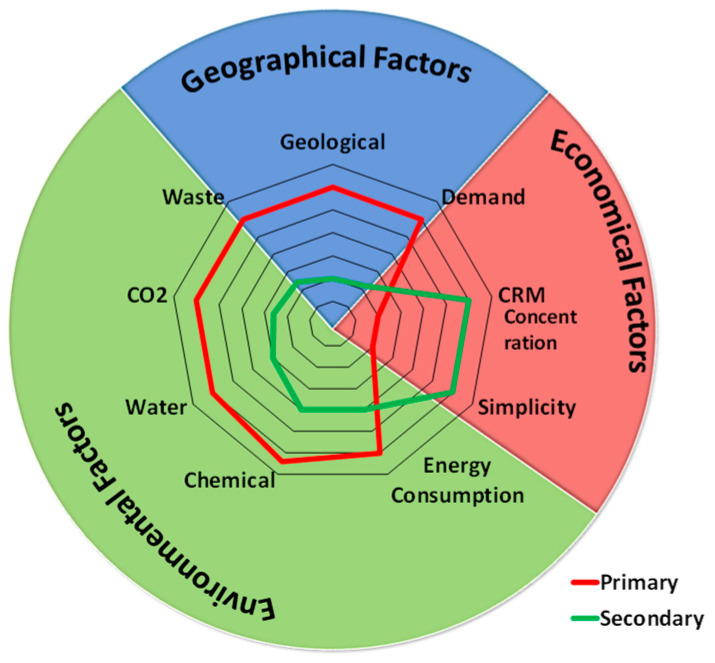
Key factors and the most impactive contributions that position the circular economy and recycling as the only sustainable solutions to address critical raw materials (CRM) and, in particular, the long-term supply constraints of rare earth elements (REE).

The sustainable solution to address the CRM and, in particular, REE supply constraints in the long run is the circular economy concept [2,3,15,16,17]. Recycling is an important strategy for meeting the challenge of this enormous increase in REE demand [11,12] due to three key factors: geographical, economic, and environmental. Figure 3 illustrates multiple contributions to these factors, showing the main impactful ones [15], which position the circular economy and recycling as the only sustainable solutions to address critical raw materials (CRM) and, in particular, the long-term supply constraints of rare earth elements (REE). The first of these factors is that recycling can be organized where end-of-life (EoL) products were used and/or collected. Thus, the production of CRMs is not limited to geological distribution, as in the case of primary ore mining. In addition, the increase in REE consumption will increase scrap availability in the next few years, thus increasing opportunities for recycling [6,17]. On the other hand, the REE concentration in secondary sources is frequently many times higher than in primary raw materials [17,18,19]. Recently, the mining of new fields with lower content of metals of interest contributes to more complicated and energy-intensive technologies for ore treatment and metal extraction. In parallel, the production of Nd–Fe–B permanent magnets consumes around 31% of REE metals [20,21], making them the most relevant type of EoL material for their recovery. The raw material costs for the production of a kilogram of sintered Nd–Fe–B are constantly increasing, now ranging from EUR 20–30 [6,7,14,15,16]. This price strongly depends on the contents of HREEs, such as Dy, Tb, and Pr, which are significantly more expensive than Nd. Thus, the overall production cost of the magnets has the potential to be significantly lower in the case of the exploitation of Nd–Fe–B scrap from end-of-life products [19,20,21,22,23]. Appropriate scrap management and standardization (including waste collection, separation, etc.) can considerably decrease and simplify secondary ore processing steps [24,25,26]. This allows radical saving of resources, leading to high economic and environmental benefits, as substantially lower energy-, time-, and chemical consumption comes together with decreased carbon emission intensity from recycling REEs than from primary production [18,19,26,27,28]. Unfortunately, landfilling is still the main choice for WEEEs with a very low recycling rate of end-of-life (EoL) REE-containing materials, as shown in Figure 1. For example, only ≈1% of Nd consumption in the EU came from recycling in 2023 [6,7]. Although research and innovation investigations continue, developing efficient and cost-effective recycling methods for permanent magnets remains a difficult task [23,24,25,27]. The necessary technology and infrastructure for recycling rare earth-containing materials are still in the early stages of development, mainly on the laboratory scale.

In recent years, the rising popularity of emerging low-carbon electric two-wheel vehicles, such as electric scooters, electric bicycles, and electric motorcycles, is likely to increase the demand for permanent magnets due to significant growth in their use and the relevant encouraging policies. According to published data [11,15,29], the increase in exploitation of Nd–Fe–B magnets in this area rose from 50% in the years before 2015 to 75% in the 2015–2020 period and 100% of the electric two-wheel vehicles produced from 2020 onwards [29,30,31]. These mobility applications include a mean magnet weight of around 270 g (typically between 60 and 350 g) [15,29,31]. Thus, this type of EoL electric motor is a continuously growing, abundant, and readily available source of spent sintered Nd–Fe–B magnets, as the average lifetime for the mentioned applications is estimated by different studies in the range of 6 years [26,27,29] or up to 15 years [31,32,33]. The collection rates of electric two-wheel vehicles in the coming years are significantly higher than those for other waste, assumed to be between 50% and 80% for the lower or upper bounds of the range [29,30,33]. As for the dismantling efficiency, its rate ranges between 75% and 90%, depending on the scenario used for calculations, i.e., also relatively high [21,24,29,30,31]. As a next step, numerous academic and industrial teams are working to reduce the quantity of REEs used in permanent magnets and to develop new technologies that are not reliant on permanent magnets; however, this is still not the reality [31,32,33].

**Figure 4:**
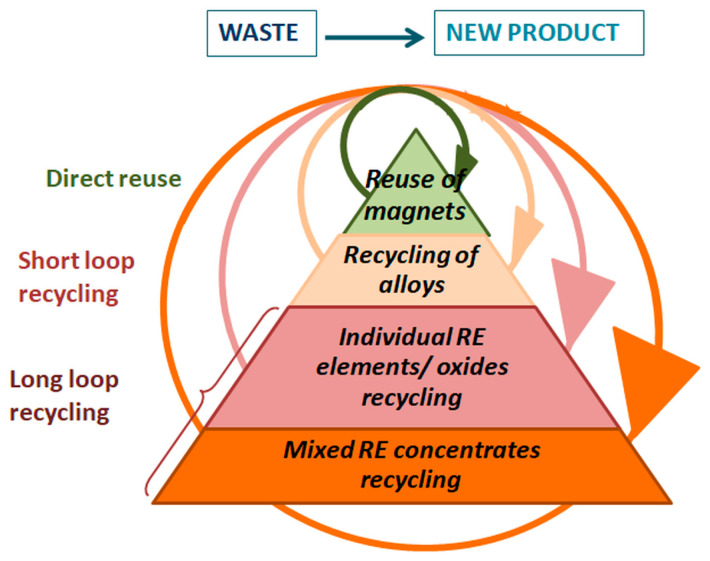
Recycling routes in the case of EoL permanent magnets and the increase in the utilized technological and economic resources, as well as the environmental and socio-economic impact, with an extended recycling loop span.

Depending on the waste classification and the methodology used, the process of closed-loop recycling can be summarized as either indirect (extended or chemical recovery of the rare earth elements) or direct reusing of materials. Figure 4 presents in a comprehensive way the recycling principles and routes in the case of EoL permanent magnets. The main factors contributing to the recycling loop span [34,35,36,37] are the quality of waste at the product’s EoL, as well as the new product requirements and further application of recycled material [18,38,39]. Technology-related challenges that establish the recycling loop length include the extraction, disassembly, and selection of magnets, hydrogen decrepitation, contamination, and magnet oxidation [40,41,42,43]. Thus, the recycling loops can be characterized by the utilized technological and economic resources (such as energy, water, time, and solvent/chemical consumption), as well as their environmental and socio-economic impacts on processing [42,43,44]. Long-loop recycling utilizes significantly more resources and has a higher environmental impact than short-loop recycling. In the case of permanent magnet recycling (Figure 4), direct reuse is magnet-to-magnet recycling, i.e., reprocessing to produce the original material or one with a comparable quality level. Studies have clearly shown that the environmental impact of magnet-to-magnet recycling is significantly lower than that of virgin magnet production [37,44,45]. Short-loop recycling or magnet-to-alloy recycling is a waste processing method used to prepare different products at a lower readiness level [46,47,48,49]. Long-loop magnet recycling or waste-to-element recycling combines waste treatment, where the thermal or chemical decomposition of materials (metallurgical or hydrometallurgical extraction and separation of REEs) leads to individual RE element/oxide extraction or the recovery of mixed RE concentrates [50,51,52]. Numerous methods for the efficient recovery of Nd from waste Nd–Fe–B magnets have been extensively investigated, with each of them offering a distinct set of advantages and concerns in Nd recovery [53,54,55,56,57]. Previous papers studied the optimization of process parameters in order to achieve more efficient, sustainable, and environmentally friendly recycling toward the preservation of strategic RE resources. Thus, it was reported that EoL permanent magnets from electric bike motors are appropriate for direct recycling due to the minimal damage of the magnet in most cases [21,27,29,30,31,32,33].

### 1.4. Direct Recycling of EoL Nd–Fe–B Magnets from Electric Bike Motors

Despite the recent political focus in Europe and across the globe on the significant economic, ecological, and strategic advantages of Nd–Fe–B magnet recycling, no industrial-scale facilities are available in our countries at the moment [6,7]. The majority of published R&D investigations on the recycling and reuse of RE-bearing magnets have studied the pre-processing steps, i.e., dismantling and extraction of magnets from the rotors, frequently without initial waste separation [46,47,48,52]. This paper presents a new recycling approach to the green and sustainable processing of non-oxidized Nd–Fe–B magnets from EoL electric bike motors. Based on precise separation, characterization, and initial treatment, standardized batches of Nd–Fe–B materials can be directly reused for the further preparation of new permanent magnets, thus maximizing recycling rates, reducing the amount of waste emissions, and minimizing the environmental impact. The reported results were obtained as activities under the project H2020 ERA-MIN3: Microwave-enhanced recovery of REEs and plastic from WEEE and reuse in additive manufacturing of novel magnetic components (MW4REMAM). In-depth recycling-oriented characterization of the scrap from EoL electric bike motors is a very important investigation stage due to the critical impact of waste properties on the recycling process, such as the elemental and phase composition, microstructure, particle size, shape, and morphology of the Nd–Fe–B material.

The elemental composition of permanent magnets differs depending on their application, the manufacturer, the year of production, etc. However, a typical Nd–Fe–B magnet consists of an (Nd, REE)_2_Fe_14_B grain phase, which is the predominant one, an Nd/REE-rich grain boundary phase (GBP), usually NdO or Nd_2_O_3_, and aminor Nd_x_Fe_y_B_z_ phase, e.g., Nd_1_Fe_4_B_4_ [58,59,60,61]. The varying composition of Nd–Fe–B magnets was demonstrated in [21,22,23,24,25,27,62]. According to previous studies, a key strategy to obtain high coercivity is the formation of continuous grain boundary layers of a non-magnetic Nd-rich phase with thickness up to several nm, surrounding Nd_2_Fe_14_B crystalline grains [63,64,65,66]. Thus, numerous grain boundary restructuring approaches have been proposed to optimize this grain boundary phase, significantly enhancing the coercivity and intrinsic corrosion resistance at low Dy consumption, to be utilized both for research investigations and industrial-scale applications [67,68,69,70,71,72].

On the other hand, different properties of the grains and GBP (such as mechanical, thermal properties, corrosion resistance, etc.) can contribute to improved extraction of RE_2_Fe_14_B magnetic grains from sintered EoL Nd–Fe–B magnets and further reuse of magnetic grains in preparation of new permanent magnets. For example, a recent investigation reported the successful extraction of a magnetic alloy by selective chemical leaching of the Nd/REE-rich phases (i.e., GBP) using a weak organic acid without compromising their magnetic performance [69]. The extracted RE_2_Fe_14_B grains have good magnetic properties, which can either be reinserted in recycled magnets or be used for novel magnet productions. The coercivity of magnets is strongly influenced by their phase composition [58,59,60], microstructure [63,64], grain size, and shape [73,74,75], as well as by the formation of a continuous grain boundary phase to isolate magnetically Nd_2_Fe_14_B grains [63,64,65,66]. Previous studies have reported on permanent magnet production using additive manufacturing (AM) or 3D printing methods [76,77,78,79,80,81,82,83]. Selective laser sintering of bonded Nd–Fe–B magnets from isotropic and anisotropic powders was reported by Mapley et al. [76]. The utilized magnetic powders had both spherical and flake particle morphologies. Experiments revealed that the produced bonded magnets possessed magnetic properties comparable to those of permanent magnets produced using other AM methods [77,78,79]. Li and co-authors demonstrated the performance of 3D-printed magnets in a DC motor configuration via back electromotive force measurements [80]. In this case, an extrusion-based additive manufacturing method was applied for the fabrication of highly dense isotropic Nd–Fe–B nylon-bonded magnets using powders with a flat plate-like morphology of magnetic particles, which had dimensions ranging from several μm up to a hundred μm. Thus, bonded magnets with better magnetic performance in the case of anisotropic particle morphology are achieved if the flake-like magnetic particles are mechanically aligned prior to consolidation.

### 1.5. Mechanochemical Approach

Mechanochemistry is a method used to induce transformations by the application of mechanical forces [84,85,86]. Since ancient times, grinding, impacting, shaking, rolling, crushing, etc., have been used in everyday life for various purposes, such as fire generation, the treatment of minerals and corn, the preparation of food, drugs, pigments, etc. Nowadays, mechanochemistry represents a breakthrough benign-by-design approach for sustainable processing and the preparation of materials based on the principles of green chemistry and engineering [87,88,89,90,91,92,93,94,95,96]. The method was applied in this study for the extraction of Nd_2_Fe_14_B magnetic grains from sintered EoL magnets for their further reuse in the preparation of new permanent magnets by AM. Mechanochemical treatment (MCT) was also expected to contribute toward refining the microstructure and the mean particle size of the studied materials [97,98,99]. Only a limited number of previous investigations on the topic can be found; however, researchers have reported the significant advantages of mechanochemistry in achieving unique material properties while working in sustainable and environmentally friendly conditions [84,85,86,87,88]. Depending on the intensity and parameters of milling, this kind of treatment can induce partial disordering, amorphization, or a decrease in the Nd–Fe–B alloy particle size down to the nanoscale [70,99,100,101,102,103]. The Nd_2_Fe_14_B hard magnetic phase can be later restored by annealing the powder, as it was previously reported [101,102,103]. Gabay and co-authors published a ball milling approach for the fabrication of nanocrystalline and amorphous RE–TM permanent magnets (where TM is a transition metal) with specific particle sizes and shapes [101]. Single-crystal nanoparticles were obtained by surfactant-assisted wet milling, and submicron-thin polycrystalline flakes were produced by a combination of dry and surfactant-assisted wet milling.

High-pressure hydrogen milling was applied by I. Dirba et al. in order to reduce the average grain size in Nd_2_Fe_14_B powders towards reaching a single-domain size [70]. The study revealed the influence of grain size and variation in Nd content on the coercivity of the treated material. Complete decomposition of the Nd_2_Fe_14_B phase into α-Fe, NdH_2_, and Fe_2_B at room temperature was registered by milling in high pressure (100 bar) in a hydrogen atmosphere. Consequent heat treatment and hydrogen desorption of the obtained powder material leads to recombination and preparation of the initial Nd_2_Fe_14_B magnetic phase, with a significant grain size reduction of nearly two orders of magnitude. Thus, the critical single-domain grain size of ≈200 nm of Nd_2_Fe_14_B was reached, and the highest H_c_ value was registered.

Simeonidis et al. performed 20 h ball milling of Nd_2_Fe_14_B powder under a protective Ar atmosphere in a mixture of heptane (as a solvent) together with oleic acid/oleylamine (as surfactants), which resulted in the preparation of 15 nm of isolated nanoparticles with improved magnetocrystalline anisotropy compared to the initial material, which had a particle size of about 40 µm. In this case, the increased duration of mechanical treatment, up to 100 h, led to the gradual formation of elongated nanoparticles followed by structural amorphization [102].

Therefore, mechanochemical treatment at appropriate conditions can give rise to a reduction in the mean particle size of the magnetic material by modifying its morphology, amorphization, or partial alloy disordering, thus significantly changing its magnetic properties. The present study is focused on the optimization of the particle size and microstructure of the investigated non-oxidized Nd–Fe–B material from EoL motorbike permanent magnets for their further reuse in the preparation of new magnets using the mechanochemical (MC) method. During the investigation, the parameters of MC treatment were varied toward the realization of the target changes in the microstructure and crystal size of the Nd_2_Fe_14_B phase, as well as to reach increased anisotropy.

Thus, the applied recycling methodology in this investigation is based on two approaches, which are ‘a priori’ aimed at increasing the efficiency and optimization of processing compared to traditional waste treatment. These include decrease in the recycling loop by the preparation of standardized batches of EoL Nd–Fe–B materials to be treated separately depending on their properties, as well as using the mechanochemical method for waste processing. The previously reported benefits of both direct recycling and mechanochemistry include a significant improvement in all technological parameters, such as energy use, climate change, and human and ecological health, together with process simplification and cost reduction. The exact evaluation of the recycling metrics will be the subject of further LCA investigation. Nevertheless, the preliminary estimation of the suggested recycling approach clearly shows that the generalized elimination of bulk solvents while maintaining safe working conditions at room temperature, the stoichiometric reactant consumption (rather than materials in excess), and simplified working conditions are the distinguishing factors that mark the superiority of mechanochemical processing over the currently utilized methods, mainly pyro- and hydrometallurgical treatment.

## 2. Materials and Methods

### 2.1. Materials

**Figure 5:**
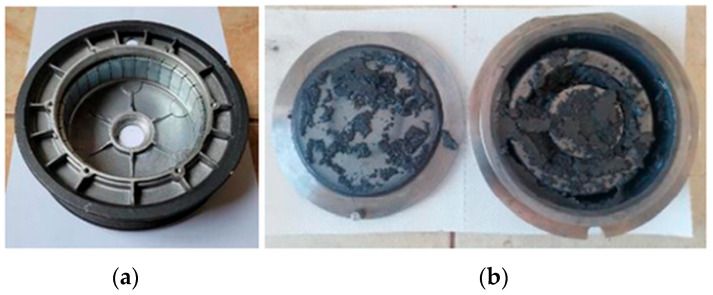
(**a**) The collected motorbike rotors. (**b**) Magnets after treatment with a vibration mill.

The present investigation is focused on the recycling of scrap magnets that were retrieved from electric bike motors. The magnets were mechanically removed from the inner part of motor rotors (see Figure 5a). After dismantling, standardized batches of spent magnets of about 1000 g were prepared for further processing; each magnet weighed about 6.4–6.5 g. The magnets were thoroughly cleaned by brushing and washing with acetone and isopropyl alcohol to remove organic adhesive and impurities caused by wear during use.

The demagnetization of the magnets was performed in an electric furnace (Vulcan, SUA, Seattle, WA, USA) at a temperature of 350 °C for 15 min under an air atmosphere. Subsequently, the magnets were allowed to cool down gradually in the furnace. The protective film (Ni in this case) was removed by scraping and brushing, and the magnets were stored in kerosene until the next experimental procedures. Prior to demagnetization, the magnets exhibited a magnetization level exceeding 430 mT. Following the demagnetization process, this level dropped to below 0.5 mT. After milling, magnetization increased from approximately 1 to 3 mT due to friction re-magnetization during milling.

### 2.2. Mechanochemical Processing

The magnets were manually crushed into small pieces (approx. 5 mm) and then milled (100 g/batch) in a vibration disk mill (RS 200, Retsch GmbH, Haan, Germany) for 10 min at 1000 rpm, with the volume of the milling jar = 250 mL, under kerosene to prevent oxidation (Figure 5b). The as-prepared material was kept and transferred for the following experiments under kerosene.

A planetary ball mill (PM100, Retsch GmbH, Haan, Germany) was used for further experiments with a vial and balls made from Y-stabilized ZrO_2_, a ball-to-powder ratio of 5:1, and a rotation speed varying from 250 to 600 rpm. The duration of planetary ball milling was changed from 15 min to 6 h.

In this study, both a vibratory disk mill and a planetary ball mill were used for the treatment of waste material. According to published data, vibratory disk mills are better suited for coarse grinding, while ball mills are more versatile and efficient for fine grinding [84,85,86,87]. According to the published papers, one of the main challenges when treating an Nd–Fe–B alloy is to avoid material oxidation during processing [99,100,101,102,103]. Thus, in the present study, MC treatment was performed without the removal of the protective organic oil (kerosene).

### 2.3. Characterization of Samples

The total metal content in the REE magnet waste was determined by ICP Spectrometry (Agilent 725 spectrometer, Santa Clara, CA, USA). Quantities used in the experiments were measured with an analytical balance. The granulation of milled magnets was determined by a vibratory sieve shaker in the domain of 90–125 µm.

Powder X-ray diffraction (XRD) patterns of samples were registered using an Empyrean diffractometer with Cu Kα radiation, 2θ= 5–80°, and a constant step of 2θ = 0.02°. Diffractograms were evaluated using the Rietveld refinement program BGMN (www.profex-xrd.org; full access date on 1 Feburary 2025), Profex 5.4.0 graphical interface [104].

Mössbauer spectra were recorded in a transmission geometry using the electromechanical spectrometer Wissenschaftliche Elektronik (Blieskastel, Germany) with a ^57^Co/Rh source (100 mCi) at a constant acceleration regime. The velocity was calibrated by the α-Fe standard [105]. The WinNormos program (http://www.wissel-gmbh.de, version from 14 July 2008) was used for the evaluation of the parameters of hyperfine interactions of each spectra component, i.e., isomer shift (IS), quadruple shift (2ε)/quadruple splitting (QS), the hyperfine effective field (B_hf_), line width (FWHM), and partial area (A). The errors for IS, 2ε, QS, and FWHM were ±0.01 mm/s. The error for B_hf_ was ±0.1 T. The computer fitting is based on the least squares method.

Microstructural and elemental composition analyses were carried out with a scanning electron microscope (SEM) (JEOL JSM 6390, Tokyo, Japan) and a state of the art modern field-emission scanning electron microscope (JEOL IT800SHL) with both in-chamber and in-lens secondary and backscattered electron detectors, coupled with two simultaneously working high-efficiency energy-dispersive X-ray detectors. The studied powders were embedded in Epofix resin inside the EPOVAC vacuum chamber. The SEM samples were polished on MD-DAC and MD-DAP sheets (Struers Pvt. Ltd., Ballerup, Denmark) using 3 and 0.25 µm diamond pastes for a smooth finish, and then washed with isopropanol. ImageJ 1.x software [106] was utilized to calculate the particle size distribution by measuring several hundreds of particles from the registered SEM images.

## 3. Results

### 3.1. Elemental Analysis

The registered chemical composition of the initial sample (Sample INI) is provided in Table 1. In addition to the main Nd–Fe–B phase elements, the composition was obtained to include some other transition metals and rare earth elements, which are intended to enhance magnet resistance to corrosion and mitigate demagnetization resulting from engine wear and elevated temperatures during operation, as well as to improve its thermodynamic and magnetic properties [32,107].

### 3.2. Mechanochemical Treatment

In this study, the tuning of Nd_2_Fe_14_B grain size and morphology was based on the changes in mechanochemical treatment parameters, thus varying the mode, intensity, and energy input. The main device used for mechanical treatment was a planetary ball mill in order, which combines moderate energy input with different actions such as impact, shear, and friction [84,85,86,87]. A Y-stabilized ZrO_2_ reactor and milling balls were used for all experiments due to the lower energy input that this material offers in comparison to the usually used steel reactors and balls. On the other hand, in case of contamination, the positive impact of a small quantity of ZrO_2_ on the magnetic properties of Nd–Fe–B was previously described [108,109]. Based on a wide-range screening of processing parameters and following the findings from previous studies [98,99,100,101,102,103], the optimum treatment parameters for the studied material were registered: rotation speed of 300 rpm and low BPWR of 5:1. These parameters were chosen for subsequent time-dependent experiments ranging from 15 min to 6 h. As a result, a region of treatment conditions was established where no degradation of the main magnetic phase of the treated material was observed, i.e., using Mössbauer spectroscopy, XRD, and SEM analysis, a 100% Nd_2_Fe_14_B phase was registered in these cases. Finding these soft-impact milling conditions, the processing time turned out to be the parameter that impacts the granulometric size, morphology, and microstructure of the main crystal phase. The optimal milling time was found to be between 30 min and 2 h for achieving the target particle size appropriate for further 3D printing of new magnets. In order to follow the effect of the performed mechanochemical processing, the properties of the initial magnetic powder (Sample INI) were compared to those of the selected key samples from the series of treated magnetic alloys. These are two samples: end-members of the experimental series, where the phase composition was preserved (mechanochemically treated for 30 min and 2 h at 300 rpm, respectively, called Sample 30 and Sample 120), and one magnetic powder with partial degradation of the main magnetic Nd–Fe–B phase (mechanochemically treated for 6 h at 600 rpm, called Sample-PD).

### 3.3. X-Ray Diffraction Analysis

**Figure 6:**
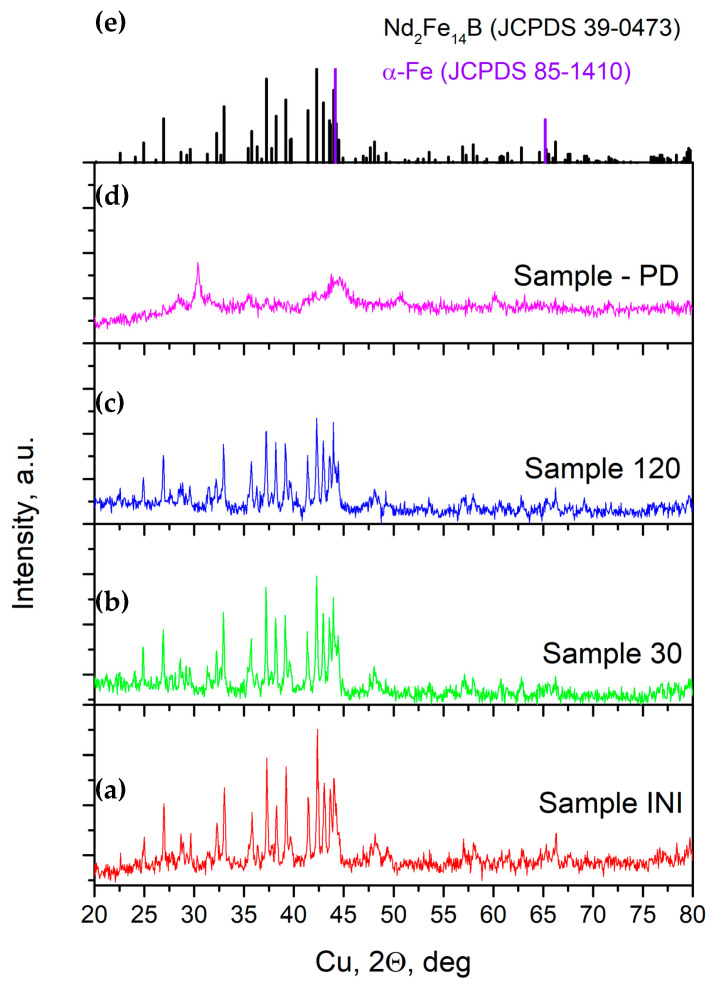
XRD patterns of the studied samples: (**a**) the initial material (Sample INI), as well as mechanically treated samples. (**b**) Sample 30, (**c**) Sample 120, (**d**) Sample PD, and (**e**) reference patterns [110].

**Figure 7:**
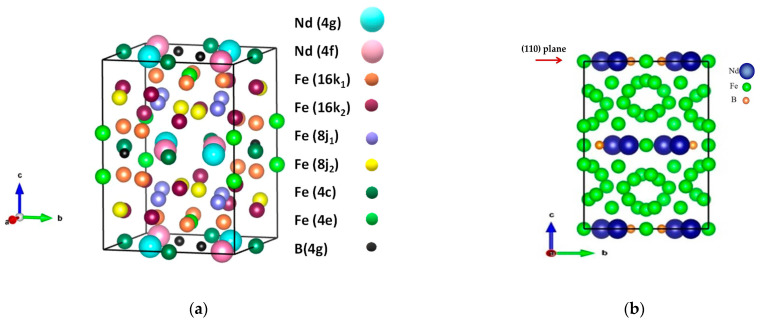
Three-dimensional visualization of the crystal structure of the tetragonal Nd_2_Fe_14_B phase using the VESTA 3 program [111]: (**a**) tetragonal unit cell of the Nd_2_Fe_14_B crystal phase and the crystallographically nonequivalent positions occupied by Nd, Fe, and B atoms; (**b**) projection of the unit cell of the Nd_2_Fe_14_B crystal showing a layer distribution of Nd, Fe, and B atoms.

Phase identification of the studied materials was carried out using XRD, and the corresponding patterns are provided in Figure 6. For the initial alloy powder and the samples treated up to 120 min (Figure 6a–c and Table 2), all the detected diffraction peaks belong to the characteristic pattern of the Nd_2_Fe_14_B only (JCPDs 39-0473) [110]. No other phases are presented in the respective diffractograms, as determined by the performed Rietveld refinement analysis. The tetragonal crystal lattice of the main phase (Figure 7a), Nd_2_Fe_14_B (space group of P42/mnm), can be described as a layer structure with an alternate stacking sequence of an Nd-rich layer and an α-Fe atom-containing plane formed by all the Fe atoms except for those occupying the 4c positions (Figure 7b) [111]. Increasing the energy input of mechanochemical treatment induces a partial decrease in the intensity and peak broadening of the reflections due to a decrease in the crystallite size, as well as the formation of strains and defects in the structure (see Figure 6). Together with the registered partial decrease in the Nd_2_Fe_14_B peak’s intensity, a change in the main peak’s intensity ratio was observed as a result of ball milling. According to previous studies, this shows an evolution of the initially randomly oriented grains on different crystallographic planes. The increase in the relative intensity ratio of the (006) and (105) peaks was reported to indicate the presence of anisotropy of the main Nd–Fe–B magnetic phase due to the texture effect, which leads to an improvement in the magnetic properties [102,109,112,113]. Further increasing the intensity or the duration of the mechanochemical processing leads to partial or complete Nd–Fe–B phase degradation. For example, in the case of material, which is mechanically treated for 6 h at 600 rpm (Sample-PD), the respective XRD pattern (see Figure 6d and Table 2) contains about 33% of the initial Nd_2_Fe_14_B phase, together with the characteristic reflections from α-Fe (JCPDS 85-1410) [110], which are easily visible. The presence of sharp lines of the respective Nd- and B-containing phases resulting from Nd–Fe–B degradation was not registered in the pattern due to their low crystallinity degree and small volume fraction. These phases contribute to an amorphous background increase together, with significant peak broadening. In addition, in parallel with the transformation of the Nd–Fe–B phase, it was registered that high-impact ball milling of material leads to contamination of the treated sample with ZrO_2_ material from the milling equipment, i.e., the milling jar and balls (JCPDS 37-1484, monoclinic and 17-0923, tetragonal) (Table 2) [110].

The average grain sizes of the Nd_2_Fe_14_B phase and lattice strain’s evolution during milling were estimated by X-ray diffraction pattern evaluation using the Rietveld refinement program BGMN, Profex graphical interface [104]. The calculated values of the contribution from these two factors were obtained and listed in Table 2. Increasing the milling duration was found to partially increase the grain size of the Nd_2_Fe_14_B phase along the c-axis. The ε values can be used for comparison of microstrains and other lattice distortions accumulated in treated material during ball milling. The obtained relatively low contribution from microstrains in the peak widths indicates that peak broadening arises mainly from particle-size considerations, i.e., from anisotropic crystallite size effects. However, the differences between anisotropic broadening caused by crystallite size and microstrains can be accurate if the Bragg peaks are well-separated and the analysis is based on single-phase data. In the case of spent magnet recycling, it is hard to extract precise information on particle size and strain due to the complicated unit cell of Nd_2_Fe_14_B, the presence of a small quantity of additional compounds (such as (REE)_2_Fe_14_B, NdO, Nd_2_O_3_, and Nd_x_Fe_y_B_z_), the formation of an amorphous phase, and the existence of a wide variety of particles with different sizes and strains (as can be seen on SEM images). The XRD patterns of the alloy powder treated various times in a planetary ball mill revealed that the initial powder consists of a tetragonal Nd_2_Fe_14_B phase, which is the magnetic matrix of the Nd–Fe–B alloy. No other crystalline phases were detected in the registered patterns with increasing milling time up to 2 h (Figure 6a–c,e). The suggested low-intensity mechanical milling (300 rpm, Zr_2_O vial and balls) leads to a change in the crystallinity and morphology of the main Nd_2_Fe_14_B phase, together with an accumulation of strains and defects. On the contrary, in the case of high-intensity mechanical milling, the initial alloy powder decomposes into nanocrystalline α-Fe phase and amorphous Nd- and B-containing compounds (Figure 6d,e).

### 3.4. Mössbauer Analysis

**Figure 8:**
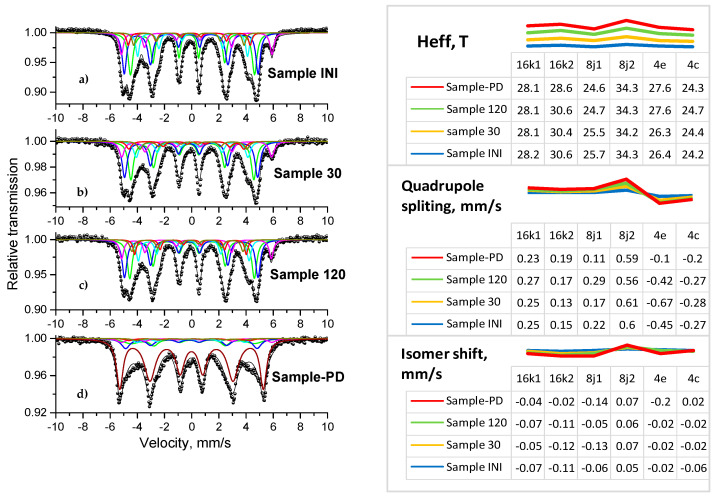
Transmission Mössbauer spectra of the studied samples: (**a**) the initial material (Sample INI), as well as mechanically treated samples: (**b**) Sample 30, (**c**) Sample 120, and (**d**) Sample PD.

Mössbauer spectroscopy is a very precise technique that provides detailed information about the iron-bearing phases present in the sample. Due to the complexity of the crystal and magnetic structure of the investigated compound, the usage of Mössbauer analysis is highly suitable to follow its local structural and magnetic properties. The tetragonal unit cell of a Nd_2_Fe_14_B crystal phase contains four formula units and 68 atoms (Figure 7a) [114,115,116,117,118]. Nd atoms occupy two crystallographically nonequivalent positions (4f and 4g). The boron atom is in the 4f position. Iron atoms occupy six crystallographically nonequivalent sites: 4e, 4c, 8j1, 8j2, 16k1, and 16k2. The hyperfine interaction of the Fe atoms occupying six crystallographic positions in a single unit cell varies due to their different chemical surroundings. The presence of six crystallographically nonequivalent iron sites gives rise to the occurrence of six individual Zeeman sextets in the room temperature Mössbauer spectrum of Nd_2_Fe_14_B, as can be seen in Figure 8a. Each component is associated with the respective crystallographic position of Fe atoms (16k1,16k2, 8j1, 8j2, 4e, and 4c) based on analysis of the crystal structure [100,114,115,116,117,118]. In this study, the six spectra components were evaluated by fitting the model for the RE_2_Fe_14_B, as suggested by numerous authors [114,115,116,117,118]. The calculated ratio of component areas fits well with the relationship of the component area on the site occupation by iron atoms, which here is 4:4:2:2:1:1. Thus, each of the subspectra was assigned to the respective iron crystallographic positions. The registered spectrum of the initial material (Figure 8a, left-hand side) contains only the characteristic Zeeman sextets of the Nd_2_Fe_14_B magnetic phase [100,114,115,116,117,118]. The evolution of material properties as a result of mechanochemical treatment can be followed in detail by the determined hyperfine Mössbauer parameters (see Figure 8, right-hand side). Due to the polycrystalline nature of the studied powders and the random orientation of the magnetization vectors in relation to the γ-rays, the ratio of the relative intensity of the lines in each sextet was assumed as 3:2:1:1:2:3 [114,115]. Lorentzian shape and equal width of the absorption lines were used for the computer fit of the spectra. Three criteria have to be followed for the best fit of this set of six quite similar sextet components of the spectrum: the same sequence of increasing B_hf_ for all the RE compounds; IS values of all subspectra are negative to an α-Fe standard, relatively; the respective iron positions must have similar QS parameters [115]. The major contribution to the hyperfine field (B_hf_) is due to the Fermi contact term. The transferred field from the iron near-neighbors increases with the increase in their number. Thus, the largest B_hf_ corresponds to the 8j2 site, which has 12 nearest neighboring Fe atoms (the widest sextet in Figure 8a, left-hand side). The obtained large QS value can be regarded as the higher electric field gradient at the site. Both 16k1 and 16k2 sites have 10 nearest neighbor Fe atoms. The value of B_hf_ of 16k1 is smaller than that of 16k2 due to the neighboring B atoms. The assignment of the 8j1 and 8j2 positions reflects their fixed proportion. The nearest neighbor Fe atoms for sites 4e and 4c are, respectively, 9 and 8. As a result, the order of B_hf_ for the six sextet components in the spectrum is as follows: 8j2 > 16k2 > 16k1 > 4e > 8j1 > 4c [116]. The spectra of treated powders were first fitted with the values of the initial sample in order to obtain a consistent set of parameters for the Nd_2_Fe_14_B based on literature data [100,114,115,116,117,118].

The computer fit of the registered Mössbauer spectra revealed the treatment conditions, where the Nd_2_Fe_14_B compound is preserved, and only the typical sixsubspectra for it were identified [100,114,115]. The evolution of the Mössbauer hyperfine parameters of each component can be followed in Figure 8 on the right-hand side. The appearance of additional components, such as α-Fe, RE–Fe_4_B_4_, and RE_2_Fe_17_, which usually coexist with the main Nd_2_Fe_14_B phase [100,114,115,116,117,118], were not detected in the spectra of materials treated at mild conditions, i.e., up to 2 h.

In this study, the appearance of new components, i.e., the partial decomposition of the main phase, as well as its transformation toward nano-sized or amorphous material, was always investigated by combining the Mössbauer spectroscopy with additional methods such as XRD and SEM. Thus, the presence of new Fe-bearing phases, such as α-Fe, iron borides (FeB or Fe_2_B), and iron oxides in the Mössbauer spectra, was registered to perform mechanical milling of the initial material with rotation speeds higher than 500 rpm and a treatment duration longer than 2 h. Both Mössbauer and XRD data revealed the appearance of about 66% soft magnetic phase α-Fe in the case of Sample PD (Figure 8d, right-hand side).

### 3.5. Scanning Electron Microscopy (SEM) and Energy-Dispersive X-Ray Spectroscopy (EDS) Analysis

**Figure 9:**
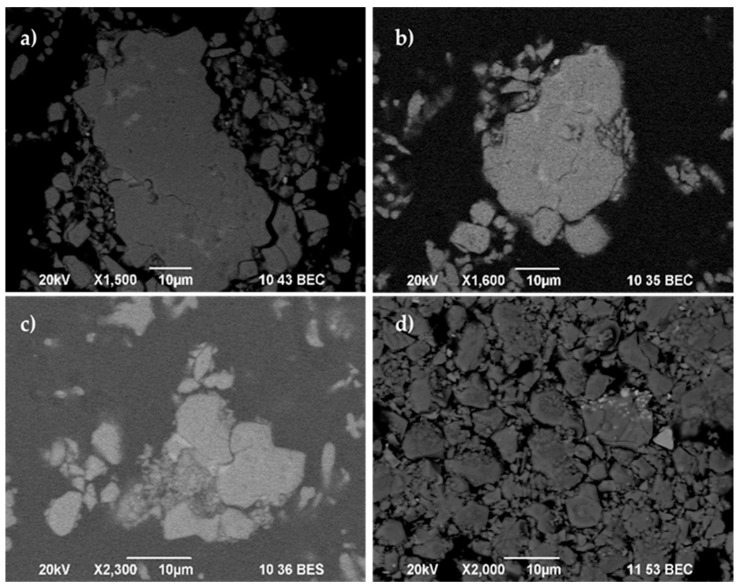
SEM micrographs of (**a**) the initial material (Sample INI), as well as mechanically treated samples: (**b**) Sample 30, (**c**) Sample 120, and (**d**) Sample PD.

**Figure 10:**
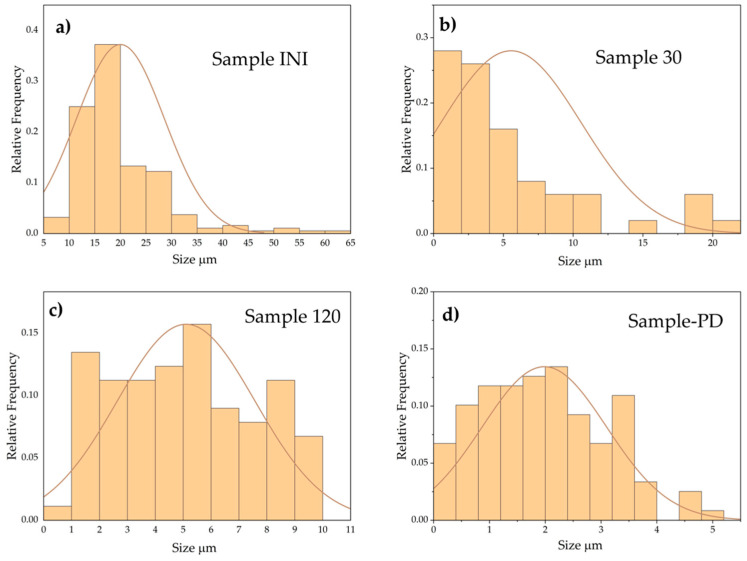
SEM micrographs showing particle size distribution in the case of (**a**) the initial material (Sample INI), as well as mechanically treated samples: (**b**) Sample 30, (**c**) Sample 120, and (**d**) Sample PD.

**Figure 11:**
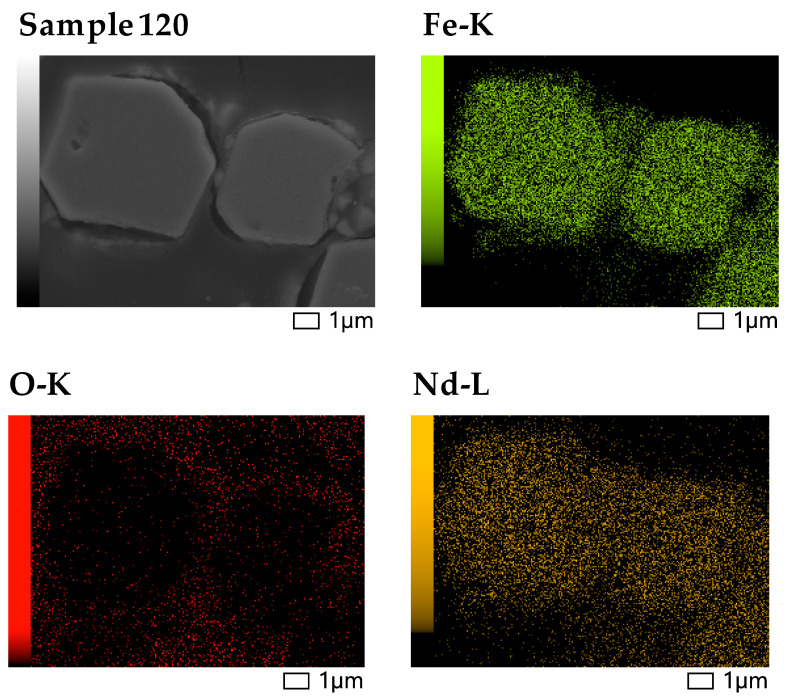
EDS analysis of iron, oxygen, and neodymium in the case of Sample 120.

**Figure 12:**
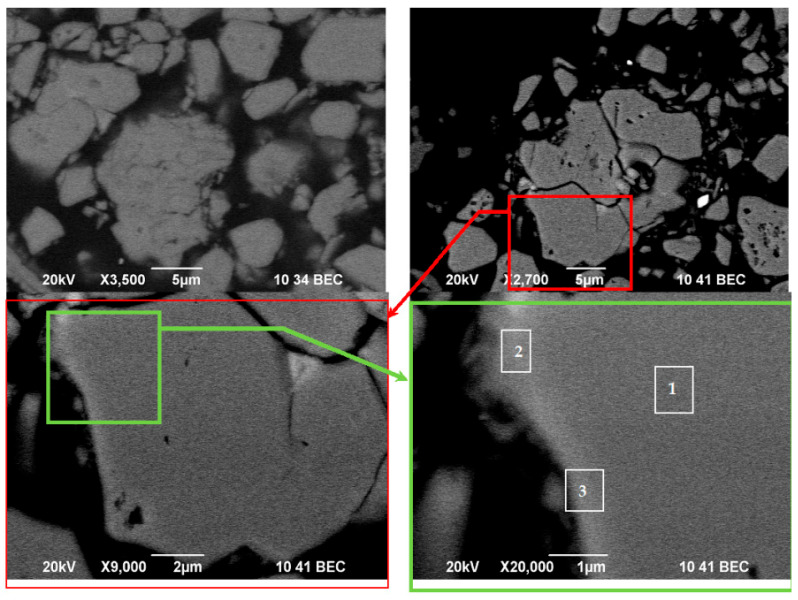
BEC images of Sample 120 at increasing magnification.

**Figure 13:**
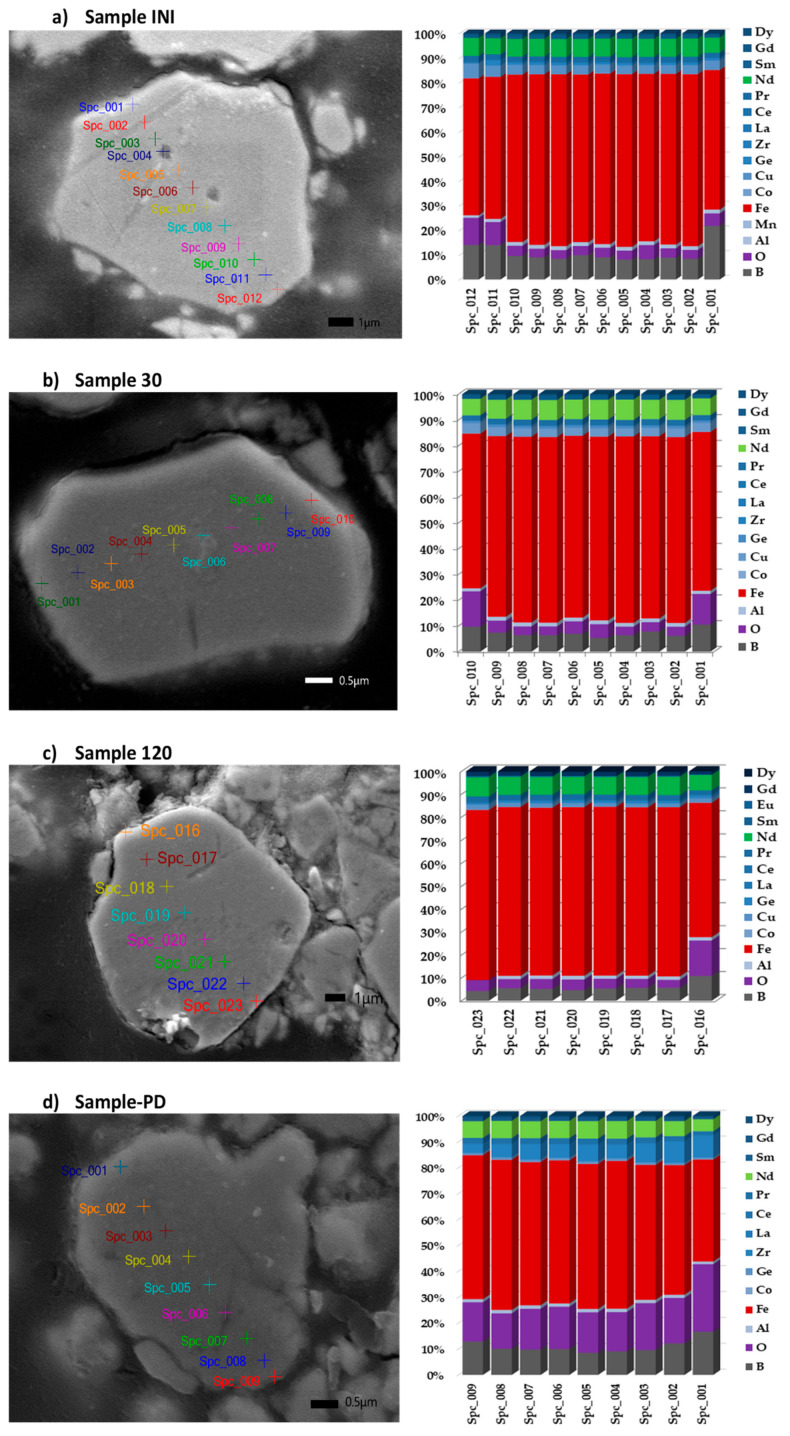
SEM/EDS line scan through a cross-section of the grains (**left-hand side**) and distribution of the elemental composition through this cross-section of the grain (**right-hand side**).

**Figure 14:**
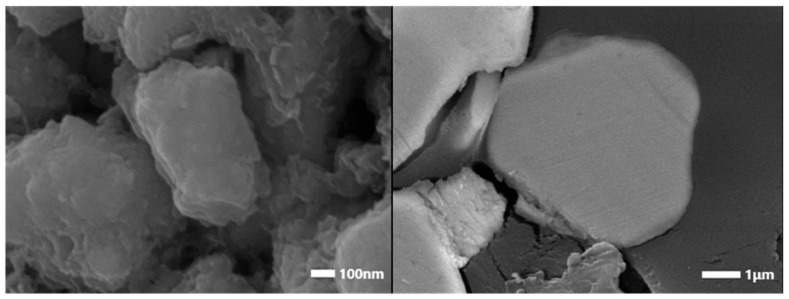
SEM analysis of Sample 120 revealing the formation of the so-called ‘river patterns’ and flake-like particles as a result of performed milling.

The registered SEM micrographs presented in Figure 9, Figure 10, Figure 11, Figure 12, Figure 13 and Figure 14 follow the transformation of the morphology, microstructure, and particle size of the analyzed materials under its mechanical treatment. Table 3 includes EDS analysis on the calculated average elemental composition of the studied materials based on the elemental maps of registered SEM images. More detailed EDS results can be seen in Table A1, where a series of analysis results are presented. The surface of the initial waste magnetic powder was registered as very rough. The fracture of the investigated material can be well seen even in the case of the initial sample as a result of pre-treatment of waste magnets using high-energy processing (vibration mill), which induced cracks in the substance (Figure 9a). Further planetary ball milling of the material with low-energy input leads to the propagation of these cracks and a change in particle size, morphology, and size distribution, as it can be followed in Figure 9 and Figure 10. The calculated particle size distribution using ImageJ software [106] is presented in Figure 10. The obtained histograms revealed the change in this distribution with the advancement of processing. The initial material contains a large variety of particles of different sizes (Figure 9a). The registered values of the statistical average particle size of treated samples showed a partial decrease and more uniform particle size distribution as a result of increasing the milling duration. An additional increase in the mechanical energy input (milling speed and duration in this case) led to a decrease in the particle size, but phase composition changed as well. EDS analysis confirmed the near-stoichiometric elemental composition of the main Nd–Fe–B magnetic phase in the case of Sample INI, Sample 30, and Sample 120, as can be seen from the values in Table 3, thus confirming the preservation of the main Nd–Fe–B phase. EDS analysis also registered no significant increase inoxygen content in these three cases. Figure 11 shows in more detail the registered EDS maps of the three main elements, i.e., iron, neodymium, and oxygen, presented in the case of Sample 120. It can be clearly seen that the grain bulk contains predominantly Fe and Nd, while the grain surface is Nd- and oxygen-enriched. REE (Nd)-rich phases can be seen in the first image due to the brighter contrast with respect to the Nd_2_Fe_14_B matrix in BSE micrographs according to the published phase identification studies [59,65,66,67,68,113]. Thus, the analysis based on both SEM images and selected area EDS maps reveals a random distribution and stoichiometric elemental composition of the main phase throughout the magnet grains, confirming the preservation of the main Nd–Fe–B phase in this sample. In parallel, Nd-rich grain boundaries are identified.

The evolution of material microstructure, including ferroelectric domains and grain boundary transformations during treatment, was followed by increasing the image magnification, as can be seen in Figure 12. Nd_2_Fe_14_B grains and the thin Nd-rich layer on the grain boundary can be clearly recognized by comparing the SEM image of the grains in a cut section of the sample used for BEC analysis (Figure 12), the respective EDS results on the elemental distribution (Table 4), as well as grain EDS mapping (Figure 11). The darkly imaged region corresponds to the Nd–Fe–B phase (spectrum 1), and the brightly imaged region is the Nd-enriched phase (spectrum 2). Bright contrast also appears along the grain surface, indicating the presence of Nd-rich thin grain surface phases (spectrum 3), as reported previously [65,66,67,68].

SEM images at higher magnification were registered for all studied samples, which revealed details about their fine microstructure (Figure 13). It can be well seen that the formation of the shell phase that covers the magnetic grains was started during the initial mechanical treatment of the material (Sample INI), and it can be barely distinguished in Figure 13a on the left-hand side. Further, mechanochemical treatment leads to the preparation of a uniform shell, which is clearly visible in Figure 13b,c on the left-hand side. The performed EDS cross-section analysis of the grains (minimum 10 EDS cross-section analyses for each sample) is illustrated in Figure 13 on the right-hand side. The registered grain elemental composition (values from EDS analysis) revealed the presence of iron, neodymium, praseodymium, and boron, and they are oxygen-free (less than 10% oxygen content in Samples INI, 30, and 120). The change in the chemical composition and formation of the REE-reach grain shell was obtained with a composition very close to that of the grain boundary phase in the sintered Nd–Fe–B. The formed surface layers are free of boron and iron but exhibit the peak of oxygen and REEs. After performing planetary ball milling (Samples 30 and 120) (Figure 13b,c), the grain size was found to have not significantly decrease. However, the formation of a sharp grain shell was registered. The latter could be the reason for the preservation and isolation of the main Nd–Fe–B magnetic grain phase. The formation of continuous layers of an Nd-rich phase (either metallic or oxide) on the surface of Nd_2_Fe_14_B grains was called “complete wetting” and was found to be beneficial for the magnetic isolation of grains [65,66,67,72]. A further increase in the mechanical energy input (e.g., Sample PD) resulted in the decomposition of the Nd–Fe–B magnetic phase. Nevertheless, in this case, some grains were found to preserve the Nd_2_Fe_14_B phase composition, having a reduced size and partially oxidized surface. An example of such grain is shown in Figure 13d, together with the registered elemental composition.

In addition, SEM analysis revealed that the performed milling of studied waste material leads to the formation of so-called ’river patterns’ and flake-like particles, as is shown in Figure 14. The registered anisotropic morphology, in particular plate- or flake-shape Nd–Fe–B particle formation, was previously found when surfactants were added during ball milling [98,99,100,101,102]. Using SEM analysis, the authors of [101] discovered that the performed milling of the studied waste RE_2_Fe_14_B material induces the formation of uniform flake-like particles with size in the range of 0.5–2 µm and thickness in the range of 7–400 nm.

## 4. Discussion

A detailed investigation of the elemental composition, phase composition, and crystallite microstructure of the initial and treated materials was performed using ICP, powder XRD, Mössbauer spectroscopy, and SEM/EDS analyses. The obtained results are presented for some key samples where the most appropriate treatment conditions were achieved. The outcomes demonstrate that the suggested mechanical processing of non-oxidized EoL Nd–Fe–B material allowed for preserving the main magnetic alloy phase, transforming its particle size, and mainly its microstructure and morphology without significant alloy oxidation. The waste processing proposed here can be exploited for the production of anisotropic Nd_2_Fe_14_B loading material with a flake form for further production of new magnets. The obtained material consists of grains with elemental composition close to the stoichiometric phase composition of the initial Nd_2_Fe_14_B compound. The formation of a thin Nd/REE-rich shell phase on the grain surface was obtained (Figure 11, Figure 12 and Figure 13). This can be explained according to the findings from previous studies. The multiphase structure of treated material (mixture of (Nd,REE)-(Fe,TM)-B alloys, (Nd,REE)-, (Fe,TM)- and B-containing compounds), as well as the anisotropy of the main Nd–Fe–B phase, induce both thermal expansion anisotropy and stress anisotropy in the ball-milled waste magnet. Detailed analysis of the Nd–Fe–B material’s microstructure under mechanical treatment [98,99,100,101,102,103,112,113] revealed different elastic properties of the Nd_2_Fe_14_B grain, which is more resistant to deformation than the Nd-rich phase. Thus, the grain boundaries can become stress-concentrated and have a high potential for crack formation during mechanical treatment. In addition, the different limits for the ductile–brittle transition of the Nd–Fe–B magnet were found for the plane parallel to the c-axis and for the one perpendicular to the c-axis [67,100,113,119,120]. Accurate investigations on the impact of the anisotropy, the accumulated energy, and the wetting mechanism previously reported on the possibilities for the parallel formation of a liquid Nd/REE-rich phase and the rearrangement of solid Nd_2_Fe_14_B grains with a temperature increase [72]. In our case, this can be induced by the local spikes of temperature and pleasure (“hot spots”) that occur during high-energy mechanical milling [84,85,86,87]. This leads to the formation of an Nd/REE-rich layer on the grain surface, which stabilizes magnetic grains and prevents their further oxidation. When the induced mechanical energy is not sufficient, the amount of liquid phase is very small, and the created Nd/REE-rich layer becomes distributed heterogeneously on the crushed grain surface (as in the case of Sample INI and Sample 30). On the other hand, ball milling experiments in this study were performed using jars and balls made of ZrO_2_. This gives rise to partial contamination of treated material (as it was registered by XRD and SEM analyses), but this was previously reported to improve the distribution of the liquid Nd/REE-rich phase significantly, and the moving speed of the grain boundaries during treatment increased in areas where the liquid phase is large [108,109]. It was concluded that making the liquid phase distribution as uniform as possible is an effective methodology for suppressing grain oxidation and aggregation [108].

In the present study, the initial crushing of magnetic waste was performed using a vibratory mill under kerosene in order to protect the material against oxidation. All the following planetary ball mill experiments were carried out without the addition of any other chemicals, which makes the proposed treatment environmentally green and sustainable. Here, the reported evolution of the main Nd_2_Fe_14_B grain phase was previously registered as a result of additive-assisted milling in the presence of a solvent and surfactants [98,99,100,101,102,103]. It was reported that the use of organic additives has multiple roles in the milling process: impeding the cold welding of crushed particles, dispersing particles, preserving the crystal structure, preventing amorphization and oxidation, and reducing contamination [102]. The two opposite events take place during the studied additive-assisted milling: the large particles break as a result of the action of mechanical forces, in parallel to an agglomeration of the newly formatted fine particles in order to minimize their surface energy. The presence of an appropriate additive and/or surfactant in the milling container was confirmed to be very efficient at impeding cold welding and the agglomeration of particles during treatment. Covering particles with additive/surfactant can lower the energy of the newly formed surfaces, thus lowering the energy required for crack propagation [84,85,86,87,102]. The formation of Nd–Fe–B nanocrystalline anisotropic flakes during milling was reported by [116]. It was obtained that at the beginning of ball milling, the internal strain in the particles increases rapidly, so the large-sized RE-Fe-B waste powders break into micrometre-sized irregular particles. Further ball milling leads to microcrack formation from an easy cleavage plane in the particles, and the microcrack cleavage expands in the grain. The development of microcracks stops reaching the edge of the grain due to crystal mismatch between grains. In the case of high-impact ball milling and a sufficiently high internal strain concentration, the microcrack could spread through the grain boundary into adjacent grains. The formation of submicrometre flakes is due to the cleavage of micron-sized particles along easy-glide (110) basal planes (see Figure 7b). In this study, the formation of micrometer flakes can be seen in the case of samples ball-milled for 2 h. Figure 14 shows the formation of so-called ’river patterns’ when the cleavage fracture occurs at the boundary of a grain with different orientations (via a step-wise process). Further cleavage of (110) planes via layer-by-layer peeling or plane splitting leads to the formation of the obtained anisotropic nanoflakes. Thus, the present study registered that even though the particle size does not significantly change, the microstructure is modified. Grains with random orientations change to the same preferred orientation [001] during ball milling. Numerous authors considered the formation of a texture as the most influential factor on the final magnet properties [32,108,109,112,113,114,116]. The use of anisotropic magnetic particles, in particular flake-shaped, for the preparation of magnets was a focus of numerous investigations both by sintering [75,76] and by additive manufacturing [77,78,79,80,81,82,83]. The studies [75,76,77,78,79,80,81,82,83] also compare the advantages and disadvantages of the use of isotropic and anisotropic Nd–Fe–B particles.

## 5. Conclusions

The presented results demonstrate that the suggested mechanical processing of non-oxidized EoL permanent magnets allowed for preserving the main Nd_2_Fe_14_B magnetic phase. The study reported the establishment of an efficient treatment approach, leading to the extraction of Nd_2_Fe_14_B magnetic grains from sintered EoL magnets. The formation of a thin layer of the Nd/REE-rich phase on the surface of the solid grains during their processing and grain rearrangement prevents considerable Nd_2_Fe_14_B alloy oxidation. The proposed waste processing can be exploited for the production of anisotropic material for further direct reuse of the magnetic phase in preparation of new permanent magnets. This approach is an environmentally friendly, sustainable, cost-effective, and scalable recycling method for magnet-to-magnet recycling. It optimizes the material value chain and significantly enhances the recycling loop of permanent magnets.

## Figures and Tables

**Table 1:** Elemental composition of the studied EoL e-bike motor magnets.

Element	Nd	Sm	Pr	Dy	Gd	La	Ce	Fe	Co	Ni	Mn	Cu
Content wt.%	17.8	0.04	4.6	<0.005	4.7	0.89	0.081	63.2	0.47	0.005	0.016	0.18

**Table 2:** Effect of the milling time on the phase composition of the treated material, as well as unit cell parameters, grain size, and lattice strain of the detected phases. XRD patterns were evaluated using the Rietveld refinement program BGMN, Profex graphical interface [104]. Registered phases are Nd_2_Fe_14_B (JCPDs 39-0473), α-Fe (JCPDS 85-1410), and ZrO_2_(JCPDS 37-1484, monoclinic and 17-0923, tetragonal) [110].

Sample	Phase Composition	Unite Cell Parameters	Mean Crystallite Size	Micro Strains
Sample INI	100% Nd_2_Fe_14_B	a = b = 0.88006 ± 0.00011 c = 1.22129 ± 0.00018 c/a = 1.38773	D(001) = 140 ± 4 nm D(100) = 82 ± 2 nm D(111) = 88 ± 2 nm	<ε^2^> = 3.8 × 10^−4^ ± 6 × 10^−5^
Sample 30	100% Nd_2_Fe_14_B	a = 0.87984 ± 0.00011 c = 1.22061 ± 0.00017 c/a = 1.38793	D(001) = 142± 5 nm D(100) = 83± 1 nm D(111) = 88 ± 1 nm	<ε^2^> = 7.2 × 10^−4^ ± 3 × 10^−4^
Sample 120	100% Nd_2_Fe_14_B	a = b = 0.88013 ± 0.00020 c = 1.22123 ± 0.00031 c/a = 1.38755	D(001) = 154 ± 7 nm D(100) = 78 ± 2 nm D(111) = 84 ± 4 nm	<ε^2^> = 11.0 × 10^−4^ ± 3 × 10^−4^
Sample PD	33% Nd_2_Fe_14_B	a = 0.88504 ± 0.00020 c = 1.22911 ± 0.00031 c/a = 1.38876	D(001) = 41 ± 5 nm D(100) = 41 ± 5 nm D(111) = 41 ± 5 nm	<ε^2^> = 9.7 × 10^−4^ ± 7 × 10^−4^
	67% α-Fe	a = b = c = 0.288 ± 0.00023	D(001) = 32± 7 nm D(100) = 32 ± 7 nm D(111) = 32± 7 nm	<ε^2^> = 12.2 × 10^−4^ ± 4 × 10^−4^
	ZrO_2_ contamination Amorphous phase			

**Table 3:** Average values of the elemental composition of the studied materials based on a registered series of EDS elemental maps for each sample. More detailed data are presented in Table A1. * nd—not detected.

Sample/Element Average wt.%	B	C	O	Mn	Fe	Co	Cu	Zr	La	Ce	Pr	Nd	Sm	Gd	Dy
Sample INI	1.86	5.98	1.29	0.07	61.81	0.62	1.38	0.00	0.64	0.07	4.79	16.22	0.24	3.62	1.41
Sample 30	1.52	5.01	3.46	0.00	62.31	0.35	1.24	nd *	0.56	0.03	4.47	16.31	0.21	3.22	1.31
Sample 120	0.98	3.96	5.43	0.03	63.52	2.25	nd	2.01	1.05	0.18	1.39	15.72	0.11	2.72	0.65
Sample PD	1.01	4.89	14.55	0.56	54.70	0.00	0.00	12.05	0.06	0.65	nd *	11.03	0.09	nd *	0.41

**Table 4:** EDS analysis on the elemental composition of Sample 120, based on EDS elemental maps of sites 1–3 in Figure 12. * nd—not detected.

Spectrum Label	O	Fe	Co	Mn	Cu	Nd	Sm	Pr	Dy	Gd	La	Ce
1	5.34	73.79	0.34	0.02	1.09	18.34	0.11	11.66	0.01	1.62	3.49	0.02
2	9.27	25.28	0.02	nd *	1.84	24.36	0.07	5.68	nd *	4.49	2.11	0.18
3	15.11	20.90	0.07	0.01	2.26	30.75	0.04	17.35	0.01	4.06	4.24	0.08

## Data Availability

The datasets presented in this article are not readily available because the data are part of an ongoing study and IP rights restrictions. Requests to access the datasets should be directed to Zara Cherkezova-Zheleva.

## Appendix A

**Table A1:** EDS analysis on the elemental composition of the studied materials based on registered EDS elemental maps of SEM images for respective samples. * nd—not detected.

Sample, wt.%	B	C	O	Mn	Fe	Co	Cu	Zr	La	Ce	Pr	Nd	Sm	Gd	Dy
Sample INI	2.27	5.64	2.66	0.01	59.55	0.27	2.53	nd *	1.07	0.15	5.13	15.8	0.09	3.16	1.27
	2.43	9.62	2.42	0.02	51.85	0.2	4.48	nd *	0.67	0.24	4.73	15.2	0.24	3.18	1.79
	1.62	4.54	1.05	0.11	59.62	0.16	3.87	nd *	0.61	0.13	4.71	16.2	0.23	3.63	1.98
	1.53	4.01	0.88	0.09	60.65	0.12	3.64	nd *	0.69	0.21	4.69	16.2	0.19	3.8	1.64
	1.42	4.09	0.88	0.05	60.76	0.13	3.46	nd *	0.54	0.09	4.71	16.3	0.19	3.84	1.78
	1.67	4.32	0.95	0.12	59.75	0.25	3.45	nd *	0.63	0.13	4.77	16.6	0.31	4.03	1.42
	1.52	4.61	0.98	0.06	60.5	0.26	3.36	nd *	0.47	0.06	4.75	16.5	0.21	3.71	1.47
	1.36	4.07	0.9	0.09	60.78	0.2	3.3	nd *	0.58	0.17	4.83	16.7	0.23	3.72	1.54
	1.4	4.53	1.45	0.05	59.59	0.11	3.4	nd *	0.63	0.18	4.89	16.6	0.32	3.57	1.73
	1.49	4.73	0.95	0.05	60.22	0.1	3.43	nd *	0.57	0.16	4.78	16.4	0.35	3.7	1.49
	1.4	5.24	0.89	0.1	60.09	0.22	3.37	nd *	0.57	0.11	4.79	16.4	0.28	3.71	1.23
	4.15	6.32	1.42	0.1	55.67	0.27	4.06	nd *	0.6	0.27	4.71	15.7	0.22	3.43	1.28
Sample 30	1.71	8.76	3.63	0	55.12	0.27	3.95	nd *	0.53	0.09	4.37	15.2	0.1	3.45	1.29
	1.25	3.93	1.16	0	61.29	0.07	3.36	nd *	0.53	0.04	4.54	16.6	0.18	3.65	1.57
	1.07	3.35	0.84	0	62.07	0.07	3.11	nd *	0.62	0.03	4.67	17.2	0.31	3.78	1.26
	1.06	3.18	0.84	0	61.94	0.14	3	nd *	0.62	0	4.71	17.3	0.31	3.79	1.5
	1.17	3.61	1.21	0	61.57	0.11	2.95	nd *	0.64	0.06	4.58	17.2	0.19	3.64	1.42
	0.89	3.54	1.32	0	61.64	0.2	3	nd *	0.66	0.15	4.43	17.2	0.24	3.76	1.3
	1.07	3.16	0.82	0	62.37	0.04	3	nd *	0.66	nd *	4.47	17.6	0.1	3.64	1.38
	1.31	3.72	0.91	0	61.47	0.06	3.14	nd *	0.49	0.09	4.86	16.9	0.26	3.72	1.43
	1.02	3.62	0.87	0	61.86	0.13	3.1	nd *	0.38	0.06	4.69	17.2	0.24	3.66	1.43
	1.77	13.18	2.97	0	53.69	0.25	3.25	nd *	0.45	0.04	4.26	14.6	0.23	3.35	0.54
Sample 120	1.87	13.13	3.97	0.63	52.3	1.32	nd *	0.02	4.48	15.57	0.17	13.4	3.52	nd *	nd *
	0.94	2.56	0.85	0.61	63.09	1.12	nd *	nd *	5.15	17.72	0.37	14.75	4.02	nd *	0.66
	0.94	2.52	0.96	0.59	62.99	1.2	nd *	nd *	5.05	17.35	0.18	11.76	4.31	nd *	0.88
	0.89	2.47	1.05	0.6	63.18	1.07	nd *	nd *	5.15	17.11	0.24	12.78	4.26	nd *	0.79
	0.77	2.68	1.15	0.68	63.05	1.14	nd *	nd *	5.08	16.94	0.28	11.66	4.3	nd *	0.62
	0.86	2.53	1.07	0.63	62.71	1.09	nd *	0.03	5.18	17.25	0.34	12.69	4.13	nd *	0.89
	0.91	2.51	0.99	0.6	63.27	1	nd *	0.01	4.95	17.51	0.34	12.84	4.11	nd *	0.63
	0.69	3.31	1.14	0.39	61.42	0.64	nd *	nd *	5.37	17.98	0.35	14.84	4.23	nd *	0.72
Sample PD	0.4	2.97	15.61	0.52	41.74	0.05	0.92	11.48	0.04	0.73	0.43	8.18	0.51	0.58	0.87
	2.25	3.87	5.32	0.55	48.77	0.00	0.03	1.49	0.05	4.45	0.22	14.38	0.78	3.31	1.48
	1.73	3.82	4.78	0.57	49.76	0.00	nd *	2.34	0.18	4.72	0.16	15.23	0.7	3.34	1.41
	1.62	3.49	3.96	0.59	53.51	nd *	nd *	2.15	nd *	4.87	0.03	16.19	0.6	3.45	1.3
	1.5	3.41	4.04	0.56	52.16	nd *	0.00	2.08	nd *	4.73	0.83	15.31	0.84	3.31	1.33
	1.8	3.87	4.31	0.56	52.54	0.00	0.00	2.04	0.1	4.96	0.99	15.24	0.73	3.29	1.34
	1.75	3.72	4.1	0.6	52.14	0.00	0.01	2.17	0.13	4.89	0.48	15.28	0.7	3.44	1.45
	1.79	3.47	12.44	0.57	52.48	nd *	0.00	10.51	nd *	0.8	0.88	15.02	0.73	nd *	1.31
	1.04	9.20	10.84	0.5	52.18	0.00	nd *	11.18	0.06	0.67	0.00	12.35	0.6	0.00	1.38
